# miR-369-3p Ameliorates Inflammation and Apoptosis in Intestinal Epithelial Cells via the MEK/ERK Signaling Pathway

**DOI:** 10.3390/ijms26094288

**Published:** 2025-05-01

**Authors:** Viviana Scalavino, Emanuele Piccinno, Gianluigi Giannelli, Grazia Serino

**Affiliations:** National Institute of Gastroenterology S. De Bellis, IRCCS Research Hospital, Via Turi 27, Castellana Grotte, 70013 Bari, Italy; viviana.scalavino@irccsdebellis.it (V.S.); emanuele.piccinno@irccsdebellis.it (E.P.); gianluigi.giannelli@irccsdebellis.it (G.G.)

**Keywords:** miRNA, IBD, MEK, intestinal epithelial cell, apoptosis, miR-369-3p

## Abstract

Inflammatory Bowel Disease (IBD) is a group of chronic and recurrent inflammatory diseases characterized by prolonged inflammation of the intestinal tract. Although it has been proven that the immune system plays a crucial role in the pathogenesis of IBD, a defective intestinal epithelium is also responsible for chronic inflammation, hence causing an over-activation of the immune response. For this reason, a therapeutic approach that acts by improving impaired intestinal homeostasis could ensure a greater therapeutic efficacy in IBD. Mitogen-activated protein kinases (MAPKs) signaling pathways may be involved in the pathogenesis of IBD. It has been demonstrated that the inhibition of mitogen-activated protein kinase kinase 1 (MEK1) may be a potential treatment against IBD since it may restore the normal epithelial function and reduce apoptosis of intestinal epithelial cells (IECs). New therapeutic strategies are emerging including small molecules such as microRNAs (miRNAs). In this study, we aimed to demonstrate that miR-369-3p was able to modulate the MEK/ERK signaling pathway. As reported by in silico analysis, miR-369-3p was capable of pairing the 3’UTR of the MAP2K1 gene. In vitro analysis demonstrated that mimic transfection with miR-369-3p in epithelial cells downregulated the expression of MEK1, reduced the activation of ERK signaling, and modulated apoptosis of epithelial cells in response to TNF-α. Moreover, miR-369-3p significantly decreased the release of pro-inflammatory cytokine IL-8. These results support the potential of miR-369-3p to prevent apoptosis of IECs, responsible for a persistent inflammatory condition in IBD, highlighting its application value in the treatment of inflammatory disorders.

## 1. Introduction

Inflammatory Bowel Disease (IBD) is a class of chronic gastrointestinal inflammatory diseases comprising Crohn’s Disease (CD) and Ulcerative Colitis (UC), typically characterized by prolonged and relapsing inflammation of the intestinal mucosa [1]. The multifactorial nature and heterogeneity of the disease has made it difficult to fully understand the mechanisms involved in the pathogenesis of IBD. Despite this, the interaction among genetic predisposition, environmental factors and inappropriate host immune activation is a crucial aspect in triggering IBD susceptibility [2].

Since IBDs are predominantly inflammatory diseases, it has been shown that the immune system is the main actor in the pathogenesis of IBD. However, the intestinal epithelium also plays a key role in inflammation by maintaining intestinal homeostasis [3]. The intestinal epithelium is the first line of defense against inflammatory triggers, and the intestinal mucosa damage associated with defective tissue healing is responsible for the aberrant immune activation and chronic inflammation distinctive of IBD [4]. It has been widely demonstrated that dysfunction of the intestinal epithelial cells (IECs) plays a key role in the IBD pathogenesis since it transmits inflammatory signals to immune cells, activating immune responses [3,5]. For these reasons, complete mucosal healing, also called “deep remission”, has become one of the goals in therapeutic advances for IBD as this provides long-term remission and thus a lower risk of requiring surgical treatment [6,7].

Mitogen-activated protein kinases (MAPKs), including the ERK, p38, and JNK families, consist of a group of intracellular serine/threonine kinases able to trigger several cellular processes including proliferation, inflammation, and apoptosis in response to different extracellular stimuli [8]. MAPKs signaling cascades are constituted by different MAPKs which act as intermediaries, starting from upstream signals and extending to downstream responses. Once activated, they catalyze the phosphorylation and then the activation of the next substrate protein. Mitogen-activated protein kinase kinase 1 (MAP2K1, also known as MEK1) is a scaffold protein that mediates the activation of the mitogen-activated protein kinase 1 (ERK) signaling pathway, which, once activated, enhanced the activation of different downstream substrates involved in cell proliferation, differentiation, viability, and apoptosis [9].

Apoptosis is a physiological mechanism of programmed cell death that ensures normal tissue turnover. In the intestine, this mechanism ensures an effective barrier function against commensal microbiota [10,11]. Damage in the intestinal epithelium due to inappropriate apoptosis causes bacterial translocation and thus chronic gastrointestinal disorders. Several studies have shown that apoptosis of IECs is overactive in the tissues of patients with UC, indicating its crucial role in the onset and progression of the disease [12]. Therefore, the inhibition of IECs apoptosis may be a good target in the clinical management of UC.

microRNAs (miRNAs) are small non-coding RNA (being on average 20–22 nucleotides in length), evolutionarily conserved and able to interact mainly with the 3′ untranslated region (3′ UTR) of target messenger RNAs (mRNAs), inducing mRNA degradation and/or translational repression [13,14]. miRNAs have a regulatory function in several biological processes including development, differentiation, proliferation, apoptosis, and immune responses. However, miRNAs have also been linked to several human diseases [15,16,17,18,19]. Over the years, miRNAs have been identified as key regulators of IBD pathogenesis since they can regulate, with positive or negative effects, the immune signaling pathways and intestinal epithelial functions [20,21,22,23]. We have previously demonstrated that the miRNA named miR-369-3p in innate immune cells was able to regulate the inflammatory responses, controlling different features of the inflammatory process [24,25,26,27].

The aim of this work was to demonstrate that miR-369-3p can act as an anti-inflammatory agent also in intestinal epithelial cells. We demonstrated that miR-369-3p modulated the expression of MEK1, reducing the inflammatory response by regulating the MEK/ERK pathway and thus decreasing epithelial cell apoptosis.

## 2. Results

### 2.1. In Silico Identification of miR-369-3p Target Gene

**Figure 1:**
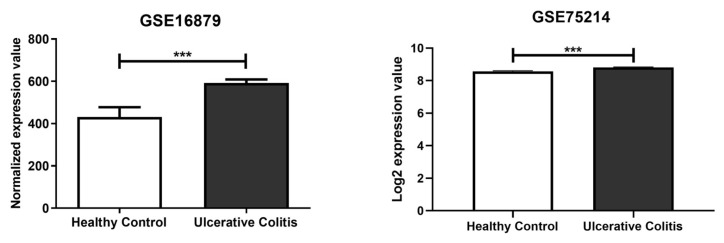
MAP2K1 expression in Ulcerative Colitis patients. Analysis of colonic tissue from UC patients and healthy controls downloaded from the GEO database (GSE16879 and GSE7524). Mean expression data are expressed as normalized expression values. *** *p* < 0.001.

In silico analysis was carried out using two different miRNA prediction tools, namely miRmap (release mirmap_202203) and DianaTools v5.0. This analysis of the mRNA target site located in the 3’ untranslated region (UTR) identified MAP2K1 as the target gene of miR-369-3p (Table S1). Subsequently, to understand whether the expression of MAP2K1 was altered in patients with UC, we analyzed the two public datasets, GSE75214 [28] and GSE16879 [29], that include 24 and 74 UC patients and six and eleven healthy controls, respectively. In the samples, the expression levels of MAP2K1 were higher in inflamed intestinal mucosa of UC patients compared with healthy controls (Figure 1; *p* < 0.001).

### 2.2. miR-369-3p Mimic Influences MAP2K1 Expression

**Figure 2:**
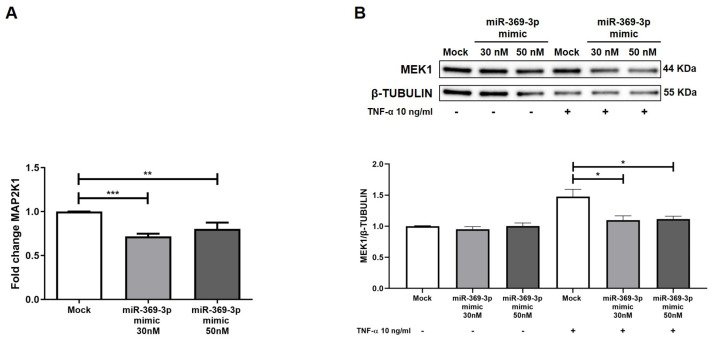
Modulation of MAP2K1 mRNA and protein expression by miR-369-3p in HT29 cells. (**A**) The mRNA expression levels of MAP2K1 were evaluated by qRT-PCR in HT29 cells transfected with miR-369-3p mimic at 30 nM and 50 nM of concentration. (**B**) Western blot analysis of MEK1 protein expression after miR-369-3p mimic transfection in the HT29 cells at 30 nM and 50 nM miRNA concentrations, both unstimulated and TNF-α-stimulated. Β-TUBULIN was used as housekeeping protein to normalize the data. Data are shown as the mean ± SEM representative of four independent experiments (* *p* < 0.05; ** *p* < 0.01; *** *p* < 0.001).

To investigate the potential modulation of MAP2K1 by miR-369-3p, we conducted, in HT29 cells, transient transfection with synthetic molecules of miR-369-3p at the concentrations of 30 nM and 50 nM. We found that the miR-369-3p mimic significantly decreased the MAP2K1 mRNA expression at both miRNA concentrations (Figure 2A; *p* < 0.01). Next, we examined the effect of transient transfection with miR-369-3p on the MEK1 protein expression levels. In a steady state, the protein expression levels of MEK1 were unchanged at both miRNA concentrations. Since the protein expression of MEK1/2 is affected by TNF-α, that was the most common inflammatory cytokine in IBD [30], we evaluated whether miR-369-3p was able to regulate the expression of MEK1 after stimulation with TNF-α. As shown in Figure 2B, the protein expression levels of MEK1 increased after TNF-α administration. However, the intracellular increase of miR-369-3p decreased the expression of MEK1 at both 30 and 50 nM concentrations compared to the treated-mock control (Figure 2B; *p* < 0.01).

### 2.3. The Modulation of MEK1 Reduces the Activation of ERK

**Figure 3:**
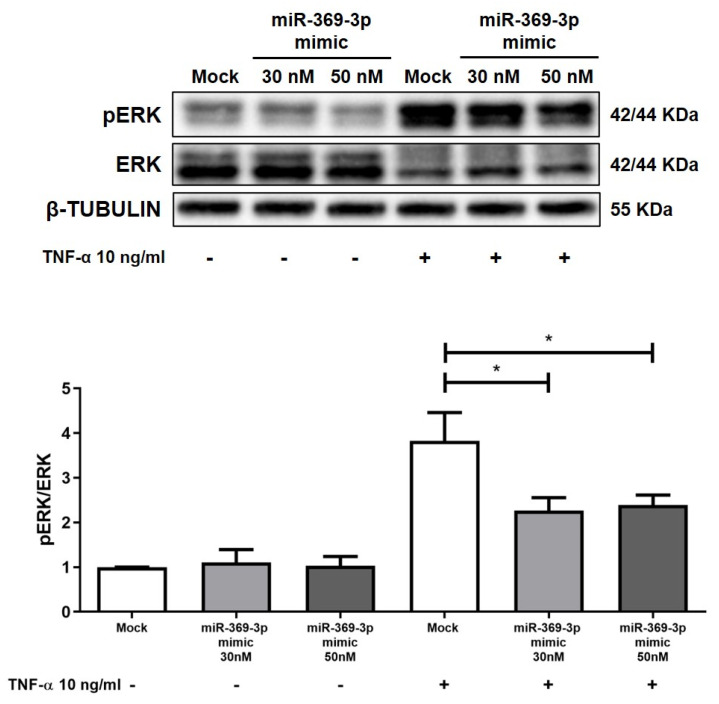
Modulation of ERK activation and expression in HT29 cells. Western blot analysis of ERK and pERK protein expression after miR-369-3p mimic transfection in the HT29 cell line without and with TNF-α stimulation. The reduction of MEK1 expression by miR-369-3p resulted in a decreased ERK activation. Β-TUBULIN was used as housekeeping protein to normalize the data. Data are shown as the mean ± SEM representative of four independent experiments (* *p* < 0.05).

Since MEK1 is responsible for the phosphorylation and thus the activation of ERK1/2 [31], we further tested the influence of miR-369-3p on the activation of the ERK protein. Western blot analysis revealed that the expression of pERK was poor in HT29 in uninflamed states, whereas stimulation with TNF-α resulted in an overactivation of pERK. Despite this, as demonstrated by the pERK/ERK ratio histogram, the rise of miR-369-3p inhibited the phosphorylation of ERK in TNF-α-stimulated cells at both the 30 and 50 nM miRNA concentrations (Figure 3; *p* < 0.01).

### 2.4. The Modulation of MEK1 by miR-369-3p Shows a Significant Impact on the Apoptosis Pathway

**Figure 4:**
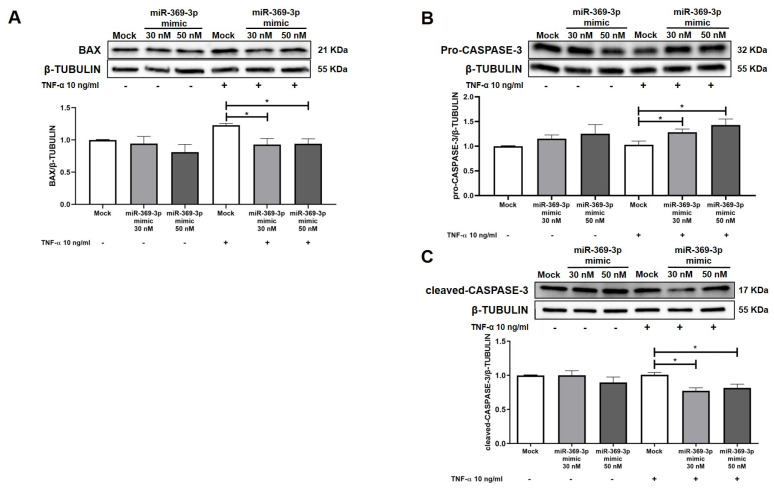
Treatment with miR-369-3p ameliorated the expression of protein involved in apoptosis in HT29 cells. (**A**) Western blot analysis of BAX protein expression after miR-369-3p mimic transfection in HT29 cells at the 30 nM and 50 nM miRNA concentrations, both unstimulated and TNF-α-stimulated. (**B**) Western blot analysis of CASPASE-3 protein expression after miR-369-3p mimic transfection in HT29 cells at the 30 nM and 50 nM miRNA concentrations, both unstimulated and TNF-α-stimulated. (**C**) Western blot analysis of cleaved-CASPASE-3 protein expression after miR-369-3p mimic transfection in the HT29 cells at 30 nM and 50 nM miRNA concentration both unstimulated and TNF-α-stimulated. β-TUBULIN was used as housekeeping protein to normalize the data. Data are shown as the mean ± SEM representative of four independent experiments (* *p* < 0.05).

ERK activation catalyzes the phosphorylation of downstream substrates which triggers the expression of many actors involved in cell death through apoptosis [32]. Hence, we tested whether miR-369-3p could modulate the process of apoptosis in HT29 cells. As shown by Western blotting, the TNF-α stimulation induced HT29 cells to express BAX, while the increase of miR-369-3p downregulated the expression level of BAX at both the 30 nM and 50 nM mimic concentrations compared to the Mock control (Figure 4A; *p* < 0.01). Moreover, we evaluated endogenous levels of inactivated CASPASE-3. In this case, we observed an increase of pro-CASPASE-3 in cells transfected with miR-369-3p followed by TNF-α stimulation compared to the Mock control (Figure 4B; *p* < 0.05). When the CASPASE-3 is activated and cleaved its expression increased inducing programmed cell death. We then assessed the expression of cleaved-CASPASE-3 after miR-369-3p mimic transfection. We found that after TNF-α stimulation miR-369-3p reduced the cleaved-CASPASE-3 at both 30 nM and 50 nM mimic concentrations in TNF-α-stimulated cells compared to the Mock control (Figure 4C; *p* < 0.05)

**Figure 5:**
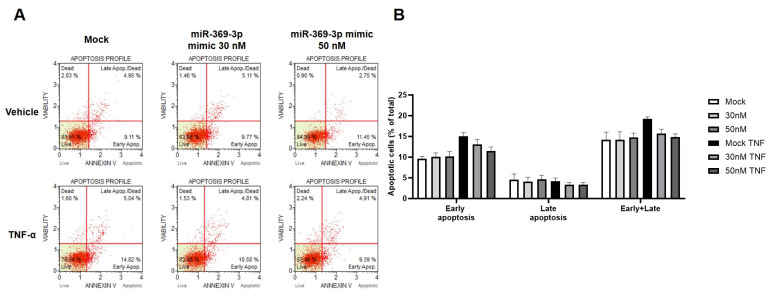
Apoptosis profile obtained using Muse Annexin V & Dead Cell Assay in HT29 cells. (**A**) Plots consist of quadrants representative of the cell populations divided into four groups (live, early apoptotic, late apoptotic, and dead) after miR-369-3p mimic transfection without and with TNF-α stimulation. (**B**) Histogram representing the percentage of apoptotic (early and late, as well as total apoptotic) cells after miR-369-3p mimic transfection without and with TNF-α stimulation. After TNF-α stimulation there was an increase of apoptotic cells compared to the steady state (*p* < 0.05). The rise of miR-369-3p decreased the percentage of apoptotic cells compared to the Mock control (*p* < 0.05). The values are shown as the mean ± SEM calculated from four independent experiments.

In addition to the protein expression analysis, we evaluated the apoptotic process using the Muse Annexin V & Dead Cell Assay kit. Compared to the steady state, stimulation with TNF-α increased the percentages of apoptotic cells, mainly early on. Despite this, the transfection with miR-369-3p significantly reduced the percentages of apoptotic cells (early and late) at both tested miRNA concentrations compared to the Mock control (Figure 5; *p* < 0.05).

### 2.5. Effect of miR-369-3p on Release of Pro-Inflammatory Cytokines

**Figure 6:**
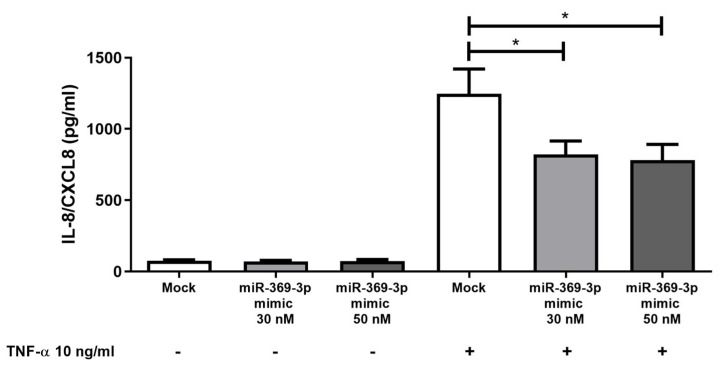
miR-369-3p modulates the release of the pro-inflammatory cytokine IL-8. miR-369-3p mimic transfection in HT29 led to a significant decrease in IL-8 at both 30 nM and 50 nM miRNA concentrations after TNF-α stimulation. Data are shown as the mean ± SEM representative of four independent experiments. (* *p* < 0.05).

The production of chemokines by intestinal epithelial cells is a key element in the pathogenesis of IBD [33]; specifically, TNF-α enhances the release of IL-8 [34,35]. We assessed whether MEK/ERK modulation by miR-369-3p was associated with a reduction of TNF-α-induced IL-8 release in HT29. We found that TNF-α stimulation was able to induce the release of IL-8/CXCL8. Moreover, compared to the Mock control, the cellular increase of miR-369-3p significantly decreased the release of IL-8 in response to TNF-α (Figure 6; *p* < 0.05).

In addition, we also evaluated the release of the two pro-inflammatory mediators of intestinal inflammation as IL-1β and CCL3. We found that the increase in IL-1β and CCL3 after stimulation with TNF-α were significantly reduced by the action of miR-369-3p mimic at both 30 and 50 nM concentrations (Figure S1; *p* < 0.05).

## 3. Discussion

The IECs play a key role in the pathogenesis and progression of UC. To date, the treatment of UC by directly targeting the intestinal epithelium remains an important focus of ongoing therapeutic development. The primary focus has been on pro-inflammatory cytokines, immune cell function, intestinal permeability, and apoptosis. The apoptosis of IECs is augmented in the colon of UC patients [36]. In fact, some evidence emphasizes the finding that an aberrant IECs apoptotic process induces intestinal damage, exacerbating chronic intestinal disorders [12,37,38]. For this reason, therapeutic approaches that operate by improving impaired intestinal functions and preventing apoptosis of IECs could be an effective therapeutic strategy in IBD. Currently, therapies for IBD involve the use of therapeutic agents that act primarily on the reduction and/or management of the inflammatory process triggered by the immune system rather than by histological healing [37]. However, these therapeutic approaches are sometimes ineffective or not tolerated. There is therefore an increasing search for new effective therapies with reduced side effects.

The MAPK signaling pathways have been implicated in the pathogenesis of IBD [8] and the inhibition of MEK1 may offer a potential therapeutic approach where the efficacy of alternative treatments is limited. A study conducted on mucosal gene expression profiles of IBD patients showed that the MAPK pathway, in particular MEK, was strongly activated in CD patients, and the application of selective MEK1/2 inhibitors could improve the pathological condition even in patients who are non-responders to anti-TNF-α biologic drugs [39]. Shi and colleagues demonstrated that treatment with N-acetyl-seryl-aspartyl-lysyl-proline (AcSDKP) played a protective role in experimental colitis and suppressed TNF-α-induced inflammatory responses of IECs by inhibiting the activation of the MEK/ERK signaling pathway [40]. In addition to the inflammatory state, the MEK/ERK pathway is involved in impaired electrolyte absorption in IBD. Zeissig and coworkers reported that the activation of ERK signaling induced by TNF-α was suppressed as a result of MEK1/2 inhibitor activity, thus increasing electrogenic sodium absorption via the epithelial sodium channel (ENaC) [30]. Moreover, natural compounds such as Qingchang Huashi and Ginsenoside were reported to have a protective function in UC. It was reported that these substances reduced the release of pro-inflammatory cytokines, promoted cell viability, and suppressed cell apoptosis via inhibition of the MEK/ERK pathway in both in vitro and in vivo UC models [41].

In this study, we have demonstrated that miR-369-3p was able to modulate the MEK/ERK signaling pathway. In our previous works, we characterized the anti-inflammatory effect of miR-369-3p in innate immune responses. We demonstrated that miR-369-3p modulated the signaling pathway of NF-kB through the modulation of C/EBP-β and NOS2 [24,42] and inhibited the assembly and activation of immunoproteasome and NLRP3 inflammasome complexes via PSMB9 and BRCC3, respectively [25,26]. Furthermore, miR-369-3p reduced the expression of PDE4B, modulating the release of pro- and anti-inflammatory cytokines via PKA and pCREB [27]. In this paper, we aimed to characterize the role of miR-369-3p in IECs. Firstly, we conducted a bioinformatic analysis to prove that MAP2K1 is a putative target gene of miR-369-3p and that altered expression of MAP2K1 would be observed in UC patients compared to healthy controls. Then, we showed that mimic transfection with miR-369-3p reduced the mRNA expression of MAP2K1 in IECs in an in vitro model. Since TNF-α is the main inflammatory mediator in IBD and is capable of triggering the overexpression of MEK1 protein, we showed that under inflammatory stimulation the rise of miR-369-3p downregulated the protein expression levels of MEK1 at 30 nM and 50 nM miRNA concentrations. In turn, MEK1 downstream mediates the activation of the ERK1/2 [31]. We showed that the inhibition of MEK1 by miR-369-3p suppressed the phosphorylation of ERK in TNF-α-stimulated cells. The MEK/ERK pathway induces the expression of many proteins involved in apoptosis.

Epithelial cell apoptosis has a significant link to the ulcerative colitis pathogenesis; in fact, it is characterized by high levels of apoptosis of IECs, which is closely connected to the severity of inflammation [38]. Several works have suggested that the production of cytokines, such as TNF-α, leads to abnormal apoptosis in IECs [43]. In the present study, our results showed that MEK/ERK modulation by miR-369-3p modulated the expression of apoptosis markers such as BAX and caspase-3 and reduced the percentage of apoptotic cells. We further explored the secretion levels of IL-8, a chemokine relevant in the onset of IBD [44]. IL-8 is a potent chemotactic factor for neutrophils which causes the aggravation of inflammation and long-term excessive infiltration [45]. In addition, we also proved that miR-369-3p was able to modulate the release of other two pro-inflammatory mediators of intestinal inflammation such as IL-1β and CCL3. Our results demonstrated that miR-369-3p was able to inhibit TNF-α-induced IL-8, IL-1β, and CCL3 expression at both miRNA concentrations. These results suggest that miR-369-3p could attenuate the inflammatory responses and apoptosis of IECs through the inhibition of MEK/ERK signaling.

In conclusion, our results showed that miR-369-3p was able to act also in IECs by reducing inflammatory signaling and the apoptotic process. This work is limited since it is still a preliminary study conducted in in-vitro models; therefore, it would be necessary to further investigate its effectiveness also in UC mice models. Nevertheless, the future application of miR-369-3p in association with other therapeutic approaches may offer a valid management strategy in UC, improving impaired intestinal homeostasis and preventing the apoptotic process of IECs, furthering the goal of ensuring better therapeutic approaches in IBD.

## 4. Materials and Methods

### 4.1. Cell Culture and miRNA Mimic Transfection

The intestinal epithelial cell line HT29 was obtained from ATCC (American Type Culture Collection, Manassas, VA, USA). The cell line was cultured in Dulbecco’s Modified Eagle Medium (DMEM, Thermo Fisher Scientific, Waltham, MA, USA) added with 10% Fetal Bovine Serum (FBS, Thermo Fisher Scientific, Waltham, MA, USA), 1% of streptomycin/penicillin, 10 mM of HEPES (Sigma-Aldrich, St. Louis, MO, USA), and 1 mM of sodium pyruvate (Sigma-Aldrich, St. Louis, MO, USA) in a 5% CO2 humidified environment at 37 °C. The HT29 cells were plated in 12 mm transwells (0.4 μm) (Corning, Corning, New York, NY, USA). When cells achieved confluence, they were transfected with miR-369-3p synthetic molecules at two concentrations, 30 nM and 50 nM (Life Technologies, Hilden, Germany) using TransIT-TKO Transfection Reagent (Mirus Bio LLC, Madison, WI, USA) in accordance with the manufacturer’s instructions. Twenty-four hours after transfection, cell cultures were basolaterally stimulated for 48 h with 10 ng/mL TNF-α (PeproTech, Rocky Hill, NJ, USA). Each transfection experiment was combined with a Mock control consisting of cells transfected with transfection reagent without miRNA molecules.

### 4.2. Total RNA Extraction and Real-Time PCR

Twenty-four hours after transfection, total RNA was obtained using TRIzol reagent (Invitrogen, Carlsbad, CA, USA) according to the manufacturer’s protocol. The RNA concentration was established using the NanoDrop spectrophotometer (Thermo Fisher Scientific, Waltham, MA, USA). Based on the manufacturer’s instructions, 1 μg of total RNAs was reverse transcribed using the iScript Reverse Transcription Supermix (BioRad Laboratories, Hercules, CA, USA). qPCR amplification reactions were performed on a CFX96 System (Biorad Laboratories, Hercules, CA, USA) using SsoAdvanced Universal SYBR Green Supermix (BioRad Laboratories, Hercules, CA, USA) and the primers MAP2K1 and GAPDH (Qiagen, Hilden, Germany). GAPDH gene amplification was used as a reference gene to obtain the relative expression of MAP2K1. The relative expression was calculated using the 2^−∆∆Ct^ method where ∆∆Ct is derived from ∆Ct of the transfected sample—∆Ct of mock condition and ∆Ct is calculated subtracting the Ct value of MAP2K1 gene from the Ct value of the GAPDH reference gene. Comparative real-time PCR was performed in triplicate.

### 4.3. Immunoblot Analysis

Twenty-four hours after transfection, cell cultures were basolaterally stimulated with 10 ng/mL TNF-α (PeproTech, Rocky Hill, NJ, USA). Forty-eight hours after TNF-α stimulation, total proteins were obtained using T-PER Tissue Protein Extraction Reagent (Thermo Fisher Scientific, Waltham, MA, USA) added with cocktail proteinase inhibitors (Sigma-Aldrich, St. Louis, MO, USA) and then quantified using Bradford’s protein assay (Biorad Laboratories, Hercules, CA, USA).

The same protein concentration for each sample was resolved by 4–20% Mini-PROTEAN TGX Stain-Free Protein Gels (Biorad Laboratories, Hercules, CA, USA) and then transferred to PVDF membranes (Biorad Laboratories, Hercules, CA, USA). The PVDFs were exposed to primary and secondary antibodies in an automated iBind Flex Western Device (Thermo Fisher Scientific, Waltham, MA, USA) according to the manufacturer’s protocol. Primary antibodies included mouse monoclonal Mek1 (#2352; Cell Signaling, Technology, Danvers, MA, USA, dilution 1:2000), rabbit monoclonal pERK (#4370; Cell Signaling, Technology, Danvers, MA, USA, dilution 1:1000), rabbit monoclonal ERK (#4695; Cell Signaling, Technology, Danvers, MA, USA, dilution 1:1000), rabbit polyclonal Bax (#2772; Cell Signaling, Technology, Danvers, MA, USA, dilution 1:1000), rabbit polyclonal Caspase-3 (#9662; Cell Signaling, Technology, Danvers, MA, USA, dilution 1:1000), and mouse monoclonal Tubulin (sc-166729, Santa Cruz Biotechnology, Inc., Heidelberg, Germany, dilution 1:1000). The secondary antibodies used were Goat Anti-mouse IgG-(H + L)-HRP conjugate (170-6516, Biorad Laboratories, CA, USA, dilution 1:500), and Goat Anti-rabbit IgG-(H + L)-HRP conjugate (31466, Invitrogen, Carlsbad, CA, USA, dilution 1:2500). Clarity or Clarity Max ECL Western Blotting Substrates (Biorad Laboratories, Hercules, CA, USA) were used to detect proteins bound to the PVDF and images were obtained using the ChemiDoc Imaging System (Biorad Laboratories, Hercules, CA, USA), analyzed with Image Lab Software version 5.2.1 (Biorad Laboratories, Hercules, CA, USA), and quantified by ImageJ Software 1.54d.

### 4.4. Annexin V Assay

Apoptosis assays on HT29 cells were performed with the Muse Annexin V & Dead Cell Kit (Cytek Biosciences, Fremont, CA, USA) according to the manufacturer’s instructions. Forty-eight hours after TNF-α stimulation, cells were collected and suspended in DMEM added with 1% of FBS. For each sample, 100 µL of cell suspension were stained with 100 µL of Muse Annexin V & Dead Cell Reagent for 20 min in the dark at room temperature and finally evaluated with the Guava Muse Cell Analyzer (Luminex Corp, Austin, TX, USA).

### 4.5. Cytokines Production

Supernatants from transfected cells were collected at 48 h after TNF-α stimulation. The analysis of IL-8, IL-1β, and CCL3 was determined using human DuoSet ELISA (R&D Systems, Minneapolis, MN, USA) according to the manufacturer’s instructions. After coating with specific capture antibody, supernatants were loaded into 96-microwell plates (Costar Corning, Corning, NY, USA). Then, the captured cytokines were detected with a detection antibody and amplified by adding a streptavidin–horseradish peroxidase conjugate. Subsequently, the reaction was enhanced using TMB (Abcam, Cambridge, UK) and the absorbance value of the samples was measured at 450 nm using the SPECTROstar Omega Spectrometer (BMG Labtech, Ortenberg, Germany).

### 4.6. Bioinformatics Statistical Analysis

The Putative target gene of miR-369-3p was identified using the miRmap (https://mirmap.ezlab.org/; accessed on 29 January 2024) [46] and DianaTools (https://dianalab.e-ce.uth.gr/microt_webserver/#/; accessed on 31 January 2024) [47] algorithms.

GraphPad Prism software version 9 was used to perform statistical analysis of data. Data are shown as the mean ± SEM, evaluated with two-tailed Student’s *t* test in accordance with a normal distribution. Data reported are representative of four independent experiments.

## 5. Patents

An Italian patent entitled “Pharmaceutical composition based on miR-369-3p as active ingredient for the treatment of chronic inflammatory disorders” (patent n° 102018000007954) was issued on 3 August 2020 to the Ente Ospedaliero Specializzato in Gastroenterologia “Saverio de Bellis”.

## Data Availability

The raw data of all western blot experiments presented in the study are openly available in FigShare at doi 10.6084/m9.figshare.28846181.

## Supplementary Materials

The following supporting information can be downloaded at: https://www.mdpi.com/article/10.3390/ijms26094288/s1.
