# Supplementary figures and images for: miR-369-3p Ameliorates Inflammation and Apoptosis in Intestinal Epithelial Cells via the MEK/ERK Signaling Pathway
Source: Int J Mol Sci. 2025 May 1;26(9):4288. doi: 10.3390/ijms26094288 (PMC12072081; doi:10.3390/ijms26094288)

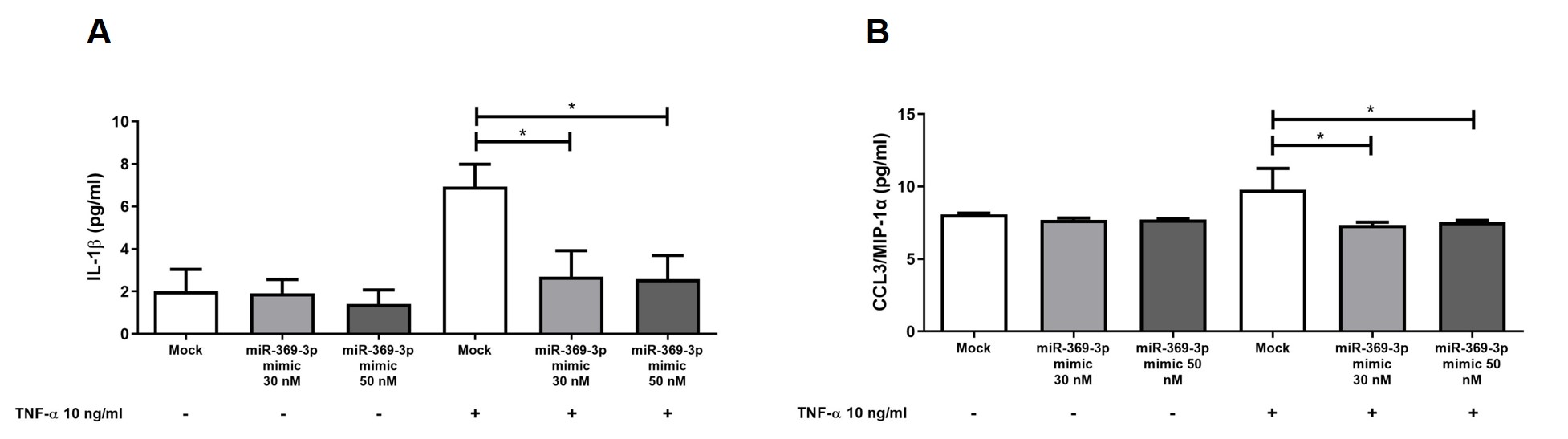

Supplement: Supplementary file 1 [file ijms-26-04288-s001.zip › Figure S1.jpg]
